# Lessons from COVID-19 mortality data across countries

**DOI:** 10.1097/HJH.0000000000002833

**Published:** 2021-03-01

**Authors:** Giovanni Corrao, Federico Rea, Gian Carlo Blangiardo

**Affiliations:** a National Centre of Healthcare Research & Pharmacoepidemiology; b Department of Statistics and Quantitative Methods, Unit of Biostatistics, Epidemiology and Public Health, University of Milano-Bicocca, Milan; c Italian National Institute of Statistics, Rome, Italy

**Keywords:** case fatality, covid-19, mortality, surveillance

## Abstract

Several online sources provide up-to-date open-access data on numbers, rates and proportions of COVID-19 deaths. Our article aims of comparing and interpreting between-country trends of mortality rate, case-fatality and all-cause excess mortality.

**Methods:**

We used data from open databases (Our World in Data mostly) for comparing mortality of eleven western countries (Austria, Belgium, Canada, France, Germany, Italy, Netherlands, Spain, Sweden, UK, USA). Between-country trends in mortality rate and case-fatality (both including deaths for COVID-19 as numerator and therefore labelled as COVID-19 mortality metrics) and all-cause excess mortality (i.e. observed deaths during the epidemic compared with those expected based on mortality in the same periods of previous years) were compared.

**Results:**

Although Belgium ranks first in mortality from COVID-19 (possibly due to the broadest criterion for attributing a death to COVID-19), it does not rank first for all-cause excess mortality. Conversely, compared with Belgium, the UK, Italy and Spain have reported lower COVID-19 mortality (possibly due to the narrower definitions for a COVID-19 death) but higher all-cause excess mortality. Germany and Austria are the unique countries for which COVID-19 mortality, case-fatality and all-cause excess mortality consistently exhibited the lowest rates.

**Conclusion:**

Between-country heterogeneity of COVID-19 mortality metrics could be largely explained by differences of criteria for attributing a death to COVID-19; in age/comorbidity structures; in policies for identifying asymptomatic people affected from SARS-CoV-2 infection. All-cause excess mortality is recommended as a more reliable metric for comparing countries.

## INTRODUCTION

There is much debate around the current availability of COVID-19 mortality data from several sources worldwide [1]. This debate is not new. What is new is the speed with which several organization have reacted to the crisis by making available data immediately accessible. Online sources such as those maintained by the WHO [2], John Hopkins University [3], European Centre for Disease Prevention and Control [4] and Our World in Data [5] provide up-to-date open-access data on numbers, rates and proportions of COVID-19 deaths. Because these data are used to inform the public and policymakers about individual and collective decisions to control the pandemic, much caution is needed when interpreting what data indicate. For example, as national governments supply data, understanding how each country manages data collection is crucial when making international comparisons. More subtly, there is also a great need to understand what data can tell us and what information they can convey, and above all the pitfalls related to bad interpretation of those data.

With these premises, taking advantage of the current interest in pandemic surveillance, this report discusses advantages and pitfalls of using some of the metrics of the impact of the COVID-19 pandemic on mortality across European and North American countries.

## INCOMPARABILITY OF COVID-19 MORTALITY DATA

At least two main types of reasons render comparisons of COVID-19 mortality between countries and over time poorly informative and potentially biased. One concerns the numerator of the COVID-19 mortality rate, that is the comparability of the criteria applied when attributing a death to COVID-19. The other concerns the denominator of the mortality rate, that is the composition of the population that the COVID-19 data were derived from.

With regard to the numerator of the COVID-19 mortality rate, according to the WHO, a death due to COVID-19 is ‘a death resulting from a clinically compatible illness, in a probable or confirmed COVID-19 case, unless there is a clear alternative cause of death that cannot be related to COVID-19 disease’ [6]. Notably however, there is large between-country heterogeneity in the reporting of COVID-19 deaths. In Russia for example, deaths are only attributed to COVID-19 based on autopsy results [7], explaining why despite having among the highest numbers of COVID-19 cases worldwide, Russia's mortality is among the lowest [8]. As well as Russia's peculiar and unique method, two other main methods of defining reportable COVID-19 deaths have been described [1]. One is based on clinical diagnosis of the cause of death, even if only suspected, irrespective of the availability of laboratory tests. Among European countries, health authority from Belgium, France, and Germany have recommended this method. Belgium has one of the broadest definitions of a reportable COVID-19 death, which includes all suspected cases, likely leading to possible overcounting relative to other countries [9]. The second, primarily based on a positive laboratory test, has been recommended from health authorities from other European countries such as Austria, Italy, the Netherlands, Spain and the UK. Particularly for this second group of countries, it follows that the availability of testing and the criteria for testing will also affect the number of COVID-19 deaths reported [1]. For example, during the first wave of the outbreak in several countries, diagnostic tests were reserved for inpatients, patients who died out of hospital with clinical features consistent with COVID-19 infection were not tested, and thus were not counted as COVID-19 deaths [10].

With regard to the denominator of the COVID-19 mortality rate, distinction should be made between mortality rate and case-fatality as respectively representing denominators of the entire population and cases with reported COVID-19 infection. Between-country demographic and clinical differences add complexity to comparisons of both mortality rate and case-fatality. Notably however, although mortality rate and case-fatality clearly increase with age, adjusting for the age distribution in a country is of weak importance when comparing western countries [11,12].

However, there is a reason for concern often ignored, which is notably important when making case-fatality comparisons. As well as involving differences in the criteria used to attribute deaths to COVID-19 and age/comorbidity structures, between-country heterogeneity also involves the policies used to identify cases of infection. In all the countries considered, policies include testing symptomatic people, including those with mild symptoms. Notably however, countries differed (and still differ) in the testing strategies used to identify asymptomatic people, for example, controls of close contacts with a confirmed case, in communities (hospitals, housing structures), in cases of hospitalization for any reason or as a personal free choice. Asymptomatic people who have been in close contact with a confirmed case are not systematically tested in Italy or the Netherlands. The definition of ‘close contact’ also differs between countries. It includes people who had contact with a case within 2 days of symptom onset in France, but up to 7 days in other countries. In Germany, Italy and Spain systematic or serial testing in a community is advised after a single confirmed case is detected, whereas in Belgium it is two cases. A personal free choice to be tested is possible against payment for asymptomatic individuals in Germany and for those who must travel in Belgium [12].

**FIGURE 1:**
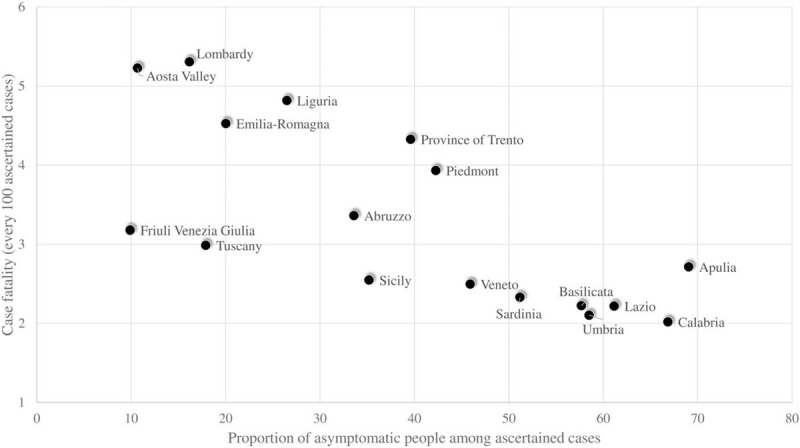
Relationship between the proportion of asymptomatic patients among reported cases and case-fatality cumulatively observed in regions of Italy. Protezione Civile. Dati COVID-19 Italia (https://github.com/pcm-dpc/COVID-19).

As well as contributing to the incomparability of COVID-19 incidence rates, these differences also strongly affect the case-fatality. In fact, it is likely that the higher the proportion of asymptomatic people is among the known cases in a given country, the better the average prognosis of an individual case is and the lower the case-fatality is in that country. Unfortunately, to the best of our knowledge, data about the proportion of asymptomatic people among known cases are only systematically reported in some countries. Because policies for identifying and ascertaining cases of infection differ between countries (at least according to official declarations) and between regions within each country, we took advantage of the availability of these data for Italian regions. As expected, an inverse relationship was observed when regressing the proportion of asymptomatic people among known cases towards the case-fatality (Fig. 1). This ecological relationship offers weak evidence to confirm our hypothesis. We cannot in fact exclude that this relationship may be partly or even fully explained by the pressure of epidemics on health systems, which both reduces the control of asymptomatic cases, and limits their capacity to meet duties of care. Regardless of the reason however, when comparing the ability of health systems to provide adequate care to patients who need them, we recommend that only symptomatic cases should contribute to case-fatality measurements.

## ALL-CAUSE EXCESS DEATHS FOR COMPARING THE SCALE OF COVID-19 IMPACT

Monitoring all-cause excess deaths (i.e. observed deaths during the epidemic compared with those expected based on mortality in the same periods of previous years [13]) is recommended by several international organizations, including the WHO [14] and the European Centre for Disease Prevention and Control [15] as a more reliable metric for comparing countries.

There are several advantages to using all-cause excess deaths. One is that as well as including deaths directly due to COVID-19 it provides a more comprehensive picture of the crisis impact, including mortality due to reduced access of timely healthcare [16–18]. Second, because all-cause excess mortality is not based on clinical diagnosis of the cause of death, incomparability due to different diagnostic criteria does not affect its value. Third, because each country is compared with itself, the comparison is not affected by between-country differences in age and comorbidity structure. Fourth, because the all-cause excess mortality rate is much larger than the COVID-19-specific mortality rate, its monitoring on a weekly basis and distribution for age, sex and social strata are less affected by random uncertainty. For example, 1.2-fold excess mortality is detectable as significant where the expected deaths are at least 200 (by accepting a type-1 error of 5% and requiring 80% power). Assuming an eight deaths per 1000 person-year mortality rate, 200 deaths occur every week in a population of 1.3 million residents. Therefore, excess mortality can be investigated on a weekly basis in almost all European and North American countries, and even in several counties/regions. In summary, all-cause excess mortality makes it possible to better understand the overall impact of COVID-19 on population health, and its specific impact on more frail groups within a population. It also facilitates tracking the impact of the pandemic in real-time, if data are reported on at least a weekly basis.

## BETWEEN-COUNTRY COMPARISONS OF MORTALITY DATA

Mortality figures across European and North American countries during the first epidemic wave are compared in Table 1. Although Belgium ranks first in mortality from COVID-19 (possibly due to the broadest criterion for attributing a death to COVID-19), it does not rank first for all-cause excess mortality. Conversely, compared with Belgium, the UK, Italy and Spain have reported lower COVID-19 mortality (possibly due to the narrower definitions for a COVID-19 death) but higher all-cause excess mortality. Germany and Austria are the unique countries for which COVID-19 mortality, case-fatality and all-cause excess mortality consistently exhibited the lowest rates.

**TABLE 1:** Summarized mortality measures of selected countries

Country	COVID-19 raw mortality rate^a^ (per 1 000 000 person-year)	COVID-19 age-adjusted mortality rate^a^^,^^b^ (per 1 000 000 person-year)	Case-fatality among COVID cases^a^ (per 100 reported cases)	All-cause excess mortality^a^ (based on expected number of deaths)
Belgium	833	321	16.1	1.27
UK	581	272	14.5	1.37
Spain	580	246	11.1	1.41
Italy	568	195	14.5	1.28
Sweden	510	193	9.9	1.20
France	451	175	14.9	1.17
United States	358	209	5.7	1.23
Netherlands	355	156	12.4	1.23
Canada	218	94	8.2	1.13
Germany	105	40	4.7	1.04
Austria	75	31	4.0	1.06

According to the coefficient of variation, heterogeneity of between-country COVID-19 mortality data is higher than that of all-cause excess mortality. The corresponding values are 0.52 for raw mortality rate, 0.50 for age-adjusted mortality rate, 0.40 for case-fatality and 0.09 for all-cause excess mortality. This indicates that with respect to COVID-19 mortality metrics, fewer sources of heterogeneity affect all-cause excess mortality.

a During the period from 02 March 2020 to 14 June 2020.

b Rates were standardized (direct method) based on the age structure of the world population (source OECD data available at https://data.oecd.org/pop/population.htm). Our World in Data (available at https://ourworldindata.org/coronavirus) and The Human Mortality Database (available at https://www.mortality.org/).

**FIGURE 2:**
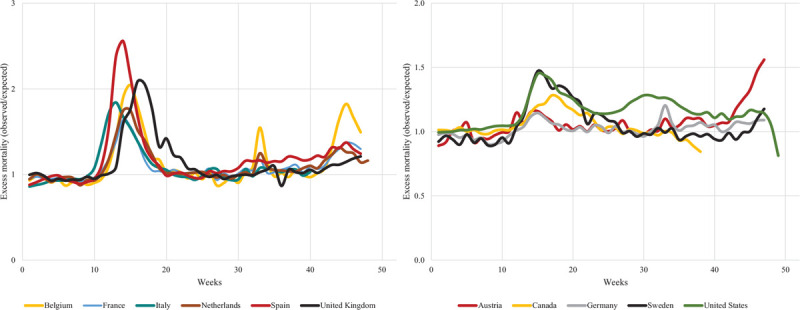
Weekly trends in all-cause excess mortality in eleven European and North American countries during the first 48 weeks of 2020. The Human Mortality Database (https://www.mortality.org/).

Figure 2 shows the entire historical series of all-cause excess mortality during the first 48 weeks of 2020 in the eleven countries considered (https://www.mortality.org/.). Two groups of countries can be distinguished in this analysis. One includes Belgium, France, Italy, the Netherlands, Spain and the UK, in which mortality in some weeks exceeded or nearly reached a two-fold greater value than expected, mainly during the first epidemic wave. In a second group that includes Austria, Canada, Germany, Sweden and the USA, mortality never exceeded a 1.5-fold greater value than expected. The sudden rise in excess mortality in Austria in the second epidemic wave is of interest. Although these figures quantify overall differences in disease between countries, excess-mortality has been shown to differ within countries, varying according to demographic parameters such as age [1], clinical parameters such as comorbidities [19] and social parameters such as specific features of different ethnic groups [20].

## SOME GENERAL WISHES FOR APPROPRIATE DATA INTERPRETATION

At present, there is no lack of data culture. Data scientists have been working for decades to provide convincing proofs on data value. Since the beginning of the pandemic, even the sceptics have understood the need for good quality data. Notably however, a great cultural effort needs to be made to reintroduce a culture of ‘information supporting decisions’ (i.e. what is called evidence-based decision-making by some) alongside that of data culture. By allowing ourselves to be solely guided by a need to quickly generate good quality data, we have forgotten that all data can be interpreted (and even manipulated with little effort [21]) in any direction, to prove any claim, no matter how outlandish.

We have heard opposing politicians claim that mortality was higher in their country than elsewhere, forgetting that they were not talking about mortality but case-fatality, and that the latter was higher for the simple fact that in that country almost all cases identified were symptomatic patients [22]. Therefore, other than placing high priority on timely collection of mortality data in the future, educational efforts to introduce (or reintroduce) good practice with regard to correctly interpreting such data should be taken into account.

## ACKNOWLEDGEMENTS

### Conflicts of interest

G.C. received research support from the European Community (EC), the Italian Agency of Drug (AIFA) and the Italian Ministry of Education, University and Research (MIUR). He took part to a variety of projects that were funded by pharmaceutical companies (i.e. Novartis, GSK, Roche, AMGEN and BMS). He also received honoraria as member of Advisory Board from Roche.

For the remaining authors, none were declared.
